# TORCH-Related Maternal Infections in a Tertiary Referral Center: Diagnostic Pathways and Prenatal Management in the Context of Canadian and European Recommendations

**DOI:** 10.3390/pathogens15101054

**Published:** 2026-10-04

**Authors:** Claudia Colomba, Nicola Serra, Costanza Giordano, Paola Di Carlo, Consolato Maria Sergi

**Affiliations:** 1 Department of Health Promotion, Mother and Child Care, Internal Medicine and Medical Specialties, University of Palermo, 90127 Palermo, Italy; claudia.colomba@unipa.it (C.C.); costanzagiordano.cg@gmail.com (C.G.); 2 Division of Pediatric Infectious Diseases, “G. Di Cristina” Hospital, ARNAS Civico Di Cristina Benfratelli, 90134 Palermo, Italy; 3 Audiology Section, Department of Neuroscience and Reproductive Sciences and Dentistry, University of Naples Federico II, 80131 Naples, Italy; nicola.serra@unina.it; 4 Infectious and Tropical Disease Unit and Sicilian Regional Reference Center for the Fight Against AIDS, AOU Policlinico “P. Giaccone”, 90127 Palermo, Italy; 5 Department of Laboratory Medicine and Pathology, University of Alberta, Edmonton, AB T6G 2R3, Canada

**Keywords:** pregnancy, congenital infections, TORCH, cytomegalovirus, toxoplasmosis, prenatal diagnosis, maternal infection, vertical transmission

## Abstract

Maternal TORCH-related infections remain a major cause of adverse pregnancy and neonatal outcomes despite advances in prenatal diagnosis and prevention. We conducted a retrospective observational study of 71 HIV-negative pregnant women referred to the Pediatric Infectious Diseases Unit of a tertiary referral center in Palermo, Italy, between June 2020 and December 2024 for suspected maternal TORCH infections. Demographic, microbiological, clinical, therapeutic, and neonatal data were retrospectively collected to characterize referral patterns, diagnostic pathways, treatment uptake, and pregnancy outcomes. Overall, 43 women (60.6%) had a confirmed maternal infection; cytomegalovirus (CMV) and *Toxoplasma gondii* accounted for most confirmed maternal infections. Maternal treatment was administered to 12/23 (52.2%) women with confirmed CMV infection and to all women with confirmed toxoplasmosis (17/17, 100%), corresponding to significantly greater treatment uptake for toxoplasmosis (*p* = 0.0008). Maternal demographic characteristics, obstetric history, treatment, and neonatal outcomes showed no significant association with trimester at referral. Three newborns were diagnosed with congenital CMV infection. Most referrals followed uncertain serological findings rather than clinically apparent maternal disease. These findings highlight the role of specialist assessment in clarifying maternal infection status, counseling patients, and informing pathogen-specific prenatal management; prospective multicenter studies are required to assess its effects on maternal and neonatal outcomes.

## 1. Introduction

Maternal infections acquired before conception or during pregnancy remain a major global public health challenge because of their potential to cause severe fetal and neonatal morbidity and mortality. Vertical transmission may occur transplacentally, intrapartum, or through breastfeeding and may lead to miscarriage, stillbirth, preterm birth, fetal growth restriction, congenital malformations, sensorineural hearing loss, neurodevelopmental impairment, and lifelong neurological sequelae. Despite major advances in prenatal care, congenital infections remain among the leading preventable causes of adverse pregnancy outcomes worldwide, particularly in low- and middle-income countries, but also in high-income settings where evolving epidemiology has created new diagnostic challenges [1,2,3,4,5].

Congenital infections have traditionally been summarized by the acronym TORCH (Toxoplasma gondii, “Others”, Rubella virus, Cytomegalovirus [CMV], and Herpes simplex virus).

Although the traditional TORCH acronym remains widely used, some authors have proposed expanded classifications that include additional congenital pathogens beyond the original TORCH components [3,4,5,6,7,8]. The TORCH framework therefore remains a useful clinical approach for evaluating maternal infections potentially associated with congenital transmission and adverse pregnancy outcomes [9,10,11,12,13,14,15,16].

Among TORCH-related pathogens, maternal cytomegalovirus (CMV) infection is widely recognized as the leading infectious cause of congenital sensorineural hearing loss and an important cause of long-term neurodevelopmental impairment in high-income countries [9,12]. Maternal *Toxoplasma gondii* infection also continues to represent an important preventable cause of congenital disease, particularly in Mediterranean regions, where environmental and dietary exposures contribute to ongoing transmission [17,18,19,20]. Recent advances in prenatal diagnosis and increasing evidence indicate that pathogen-specific interventions have improved opportunities for risk stratification, counseling, and prenatal management [11,12,18,19]. Consequently, national and international guidelines emphasize timely recognition of maternal infection, accurate diagnostic assessment, and appropriate follow-up during pregnancy [12,21,22,23,24].

Despite these advances, pregnant women are frequently referred to tertiary infectious disease centers because of inconclusive serological findings, uncertainty in laboratory result interpretation, suspected fetal involvement, or the need for specialist counseling [12,16,17,25,26]. In such cases, diagnostic evaluation often requires integration of epidemiological data, maternal clinical history, serological and molecular testing, and fetal imaging findings to distinguish active infection from previous immunity, persistent antibody responses, or false-positive results [12,16,17]. This diagnostic complexity may delay clinical decision-making, increase maternal anxiety, and affect adherence to recommended investigations and follow-up strategies [25,26,27].

Although current guidelines provide detailed recommendations for the diagnosis and management of congenital infections, real-world evidence describing referral patterns, diagnostic pathways, treatment uptake, and pregnancy outcomes in specialized referral settings remains limited [9,10,28,29,30]. Improved characterization of these processes may help identify barriers to timely diagnosis and management and support the optimization of clinical care pathways for maternal infections during pregnancy.

Therefore, this retrospective observational study included HIV-negative pregnant women referred to a tertiary pediatric infectious diseases unit because of suspected maternal TORCH-related infections. The objectives were to characterize referral patterns; describe diagnostic pathways; evaluate uptake of recommended investigations and treatment; and report available maternal, pregnancy, and neonatal outcomes.

## 2. Materials and Methods

### 2.1. Study Design and Setting

This retrospective observational study was conducted at the Pediatric Infectious Diseases Unit of ARNAS Civico Di Cristina Benfratelli Hospital, Palermo, Italy, a tertiary referral center for the diagnosis and management of congenital and perinatal infections between June 2020 and December 2024. The outpatient clinic acts as the regional referral center for the diagnostic evaluation and follow-up of pregnant women with suspected maternal infections potentially associated with congenital transmission. The Unit provides multidisciplinary care for pregnant women with confirmed or suspected maternal infections in collaboration with obstetricians, microbiologists, virologists, fetal medicine specialists, radiologists, and neonatologists.

### 2.2. Study Population

**Figure 1:**
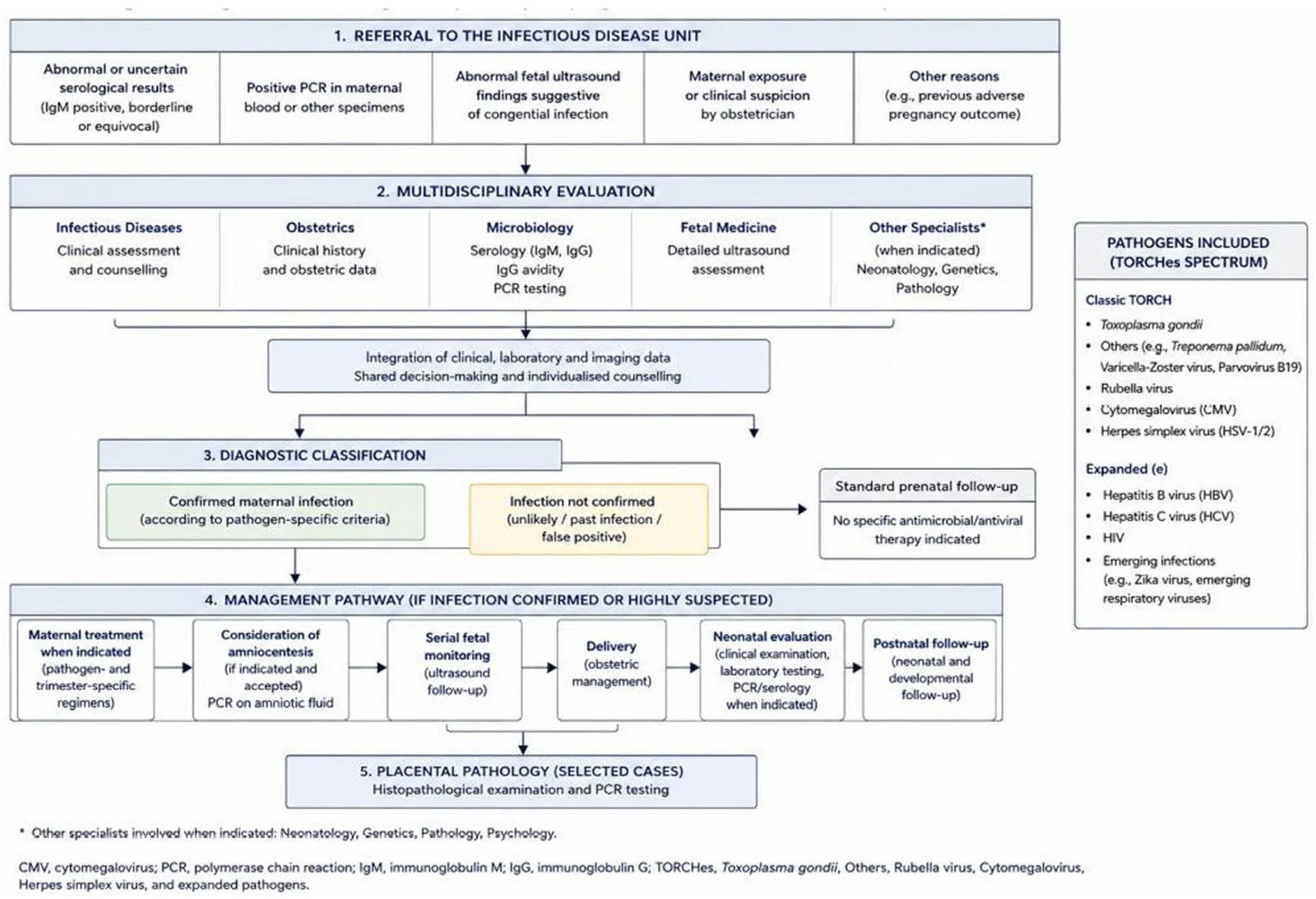
Diagnostic and management pathway for pregnant women referred for suspected TORCH infections.

A total of 71 HIV-negative pregnant women were consecutively included. The median maternal age was 30.0 years (mean, 29.4 ± 5.6 years; range, 16–45 years). Pregnant women were not recruited through universal antenatal screening but were consecutively referred to our tertiary Pediatric Infectious Diseases outpatient clinic by obstetricians or primary care physicians because of clinical, epidemiological, serological, molecular, and/or fetal ultrasound findings raising suspicion of a maternal infection potentially associated with congenital transmission. Women with known HIV infection at referral or in whom HIV infection was identified during follow-up were excluded because they required a distinct diagnostic and therapeutic pathway. Women were considered adherent to follow-up when they attended the scheduled specialist evaluations and completed the recommended clinical, laboratory, and diagnostic investigations throughout pregnancy until delivery. Women who did not adhere to the recommended follow-up or had insufficient clinical or laboratory data for diagnostic classification were excluded. As shown in Figure 1, women were referred to the Infectious Diseases outpatient clinic for suspected maternal TORCH infection when clinical, epidemiological, laboratory, and/or fetal ultrasound findings raised concerns of a maternal infection potentially associated with congenital infection [4,6,13,15,22,23,24,25,26,31].

The TORCH pathogens considered in this study were Toxoplasma gondii, *Treponema pallidum*, rubella virus, cytomegalovirus, herpes simplex virus, varicella-zoster virus, and hepatitis B and C viruses. Other infections, including parvovirus B19, were also assessed when clinically or epidemiologically indicated. The referral criteria and diagnostic approach remained consistent throughout the study period (June 2020–December 2024), while the selection of pathogen-specific investigations also considered evolving epidemiological patterns and geographical exposure, together with the clinical presentation, maternal history, laboratory findings, and/or fetal ultrasound abnormalities. The subsequent diagnostic assessment followed a multimodal, pathogen-specific approach, integrating maternal clinical and epidemiological history with pathogen-specific serological and molecular investigations and, when clinically indicated, fetal ultrasound findings and invasive prenatal diagnostic procedures. Management was based on institutional protocols and national and international guidelines. A maternal infection was considered confirmed when the diagnostic assessment, supported by pathogen-specific confirmatory laboratory testing, provided sufficient evidence of active or recent maternal infection according to established national and international diagnostic recommendations [2,4,7,12,18,19,23,31,32]. The pathogen-specific diagnostic algorithms, including first-line maternal investigations, confirmatory testing, the criteria used to confirm maternal infection, and subsequent fetal and neonatal assessment, including ultrasound, amniotic fluid testing, and postnatal follow-up when indicated, are summarized in Table 1.

### 2.3. Data Collection

Clinical, laboratory, and electronic medical records were retrospectively reviewed from the specialized ambulatory service for pregnancy-related and maternal–fetal infections within the pediatric branch of ARNAS Civico; this ambulatory service is managed by the Pediatric Infectious Diseases Unit located at the Ospedale dei Bambini “G. Di Cristina”. Collected variables included maternal age, nationality, gestational age at first infectious disease (ID) consultation referral, suspected infectious agent, serological and molecular diagnostic results, trimester of diagnosis, maternal treatment, adherence to follow-up, indication for invasive prenatal procedures, pregnancy outcome, neonatal microbiological findings, and neonatal clinical outcome. Pregnancy trimesters were defined as follows: first trimester, up to 13 weeks and 6 days; second trimester, from 14 weeks and 0 days to 27 weeks and 6 days; and third trimester, from 28 weeks and 0 days onward.

All patients included in this retrospective study had provided written informed consent for the collection, storage, and processing of their clinical data through the standardized ARNAS pre-hospitalization consent procedure routinely used for clinical care and data management, in accordance with institutional policies and applicable ethical and regulatory requirements [33]. Because of the retrospective observational design, all data were anonymized before analysis, and patient confidentiality was maintained throughout the study.

### 2.4. Statistical Analysis

Categorical variables were summarized as frequencies and percentages, whereas continuous variables were reported as mean ± standard deviation (SD) or median and interquartile range (IQR [Q1–Q3]), as appropriate.

Associations between categorical variables were assessed using the chi-square test or Fisher’s exact test, as appropriate.

The normality of continuous variables was evaluated using the Shapiro–Wilk test. Comparisons of continuous variables among pregnancy trimesters were performed using one-way ANOVA when data were normally distributed. When the ANOVA test was significant (*p* < 0.05), post hoc pairwise comparisons were performed using Scheffé’s method. For non-normally distributed continuous variables, comparisons were performed using the Kruskal–Wallis test.

All tests were two-sided, and *p*-values < 0.05 were considered statistically significant. Statistical analyses were performed using MATLAB R2008b (MathWorks, Natick, MA, USA).

## 3. Results

Table 2 summarizes the demographic, obstetric, and clinical characteristics of the study population.

The demographic, obstetric, and clinical characteristics of the study population are summarized in Table 2. A total of 71 HIV-negative pregnant women were included. Seventy women were born and resided in Italy, whereas one woman was born in Nigeria and had been living in Italy for approximately five years at the time of referral. Overall, 43 women (60.6%) were classified as having a confirmed maternal infection following the diagnostic assessment. CMV and Toxoplasma gondii accounted for most confirmed diagnoses. Pathogen-specific treatment was administered in 30 of the 43 confirmed cases (69.8%). Complete follow-up until delivery was available for 65 women, and three newborns presented findings consistent with congenital CMV infection.

In the first analysis, we stratified our sample according to trimester of pregnancy to investigate possible associations with maternal age, primigravidity, confirmed maternal infection, treatment in confirmed cases, complete follow-up until delivery, and newborns with signs of infection. The results are shown in Table 3.

**Figure 2:**
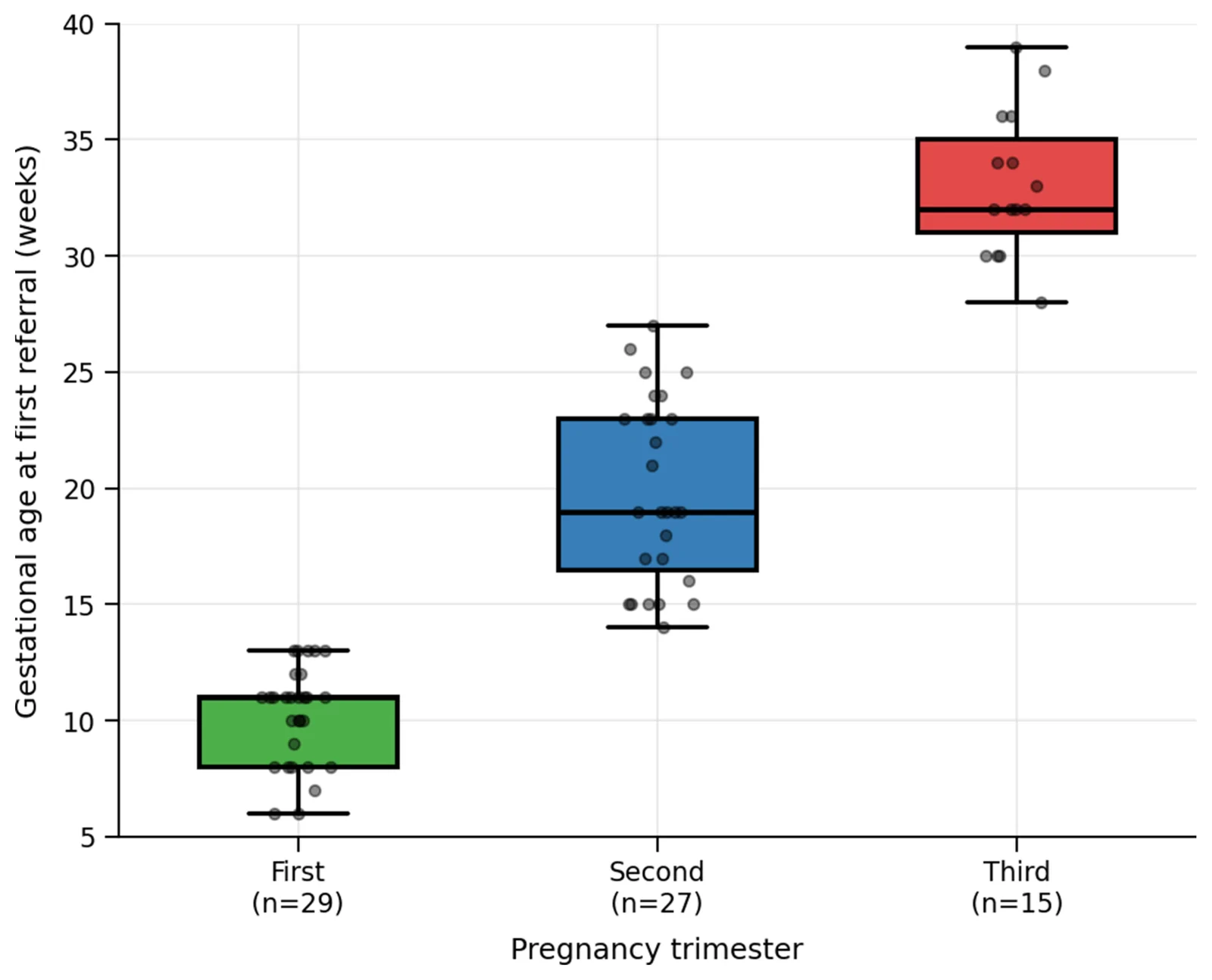
Gestational age at first infectious disease consultation. Note: Boxes represent the interquartile range (IQR), the horizontal line indicates the median, whiskers represent the minimum and maximum values, and circles indicate outliers.

No significant associations were observed for maternal age, primigravida women, confirmed maternal infection, treatment administration, follow-up completion, or neonatal infection outcomes. The distribution of referral timing is illustrated in Figure 2.

An additional analysis was performed by stratifying our sample into two groups: pregnant women who received and did not receive treatment.

Table 4 summarizes the comparison between women who received treatment and those who did not. As expected, treatment was administered only to women with confirmed maternal infections. The distribution of infection types differed between groups, with CMV more frequently represented among untreated women and a greater proportion of confirmed Toxoplasma gondii infections among treated women. All women with confirmed toxoplasmosis received treatment (17/17, 100%), compared with 12/23 (52.2%) women with confirmed CMV infection (Fisher’s exact test, *p* = 0.0008). The remaining comparisons are presented descriptively and should not be interpreted as evidence of treatment effects.

## 4. Discussion

This real-world study describes the spectrum of suspected TORCH-related maternal infections evaluated in a tertiary referral center and the recorded diagnostic and therapeutic decisions in routine clinical practice (Supplementary Table S1). Referrals were driven mainly by uncertain laboratory findings rather than clinically evident maternal disease. None of the women had significant clinical manifestations at specialist evaluation; most were referred because of positive or equivocal serological results that required further interpretation. More than half were ultimately classified as having a confirmed maternal infection, whereas the remaining women did not meet the pathogen-specific criteria after completion of the diagnostic assessment [12,13,14,34].

Interpretation of maternal history, gestational age, serology, molecular testing, fetal imaging, and repeat laboratory assessment may help distinguish primary infection from previous immunity, persistent IgM positivity, false-positive results, and other sources of laboratory uncertainty [12,13,15,16,17,18].

Cytomegalovirus represented the leading indication for referral and the most frequent confirmed diagnosis, consistent with the current epidemiology of congenital infections in high-income countries. The predominance of CMV underlines the continuing need for early diagnosis, standardized counseling, and timely referral to specialist centers. Although antiviral treatment has become increasingly supported by recent evidence, therapeutic uptake in our cohort remained lower than that observed for toxoplasmosis. This difference probably reflects persistent uncertainty regarding maternal perception of treatment benefit, concern about drug exposure during pregnancy, and the complexity of counseling surrounding congenital CMV [12,29,34,35,36].

Treatment uptake was significantly higher among women with confirmed Toxoplasma gondii infection than among those with confirmed CMV infection (17/17, 100% vs. 12/23, 52.2%; Fisher’s exact test, *p* = 0.0008). This finding may reflect the long-standing implementation of screening programs and well-established therapeutic algorithms in Italy [18,37,38].

This observation may also reflect the epidemiological and cultural context of Mediterranean countries [17,18,19,20,37,38], where awareness of toxoplasmosis during pregnancy has been promoted for several decades through routine prenatal counseling and public health recommendations. As a result, pregnant women may be more familiar with the risks associated with Toxoplasma gondii infection and therefore more likely to accept antimicrobial treatment [18,19].

By contrast, congenital cytomegalovirus has only recently received comparable attention in prenatal care, and uncertainty regarding the benefits of maternal antiviral treatment may still influence treatment acceptance.

The timing of referral to the infectious diseases service differed according to pregnancy trimester, reflecting the gestational age at which women were evaluated within routine prenatal care pathways.

In our cohort, referrals occurred across all trimesters of pregnancy. However, the retrospective design of the study does not allow evaluation of the factors influencing referral timing, and no conclusions can be drawn regarding referral pathways, risk-based referral strategies, or the appropriateness and timeliness of specialist assessment.

Early diagnosis remains particularly relevant for congenital cytomegalovirus and toxoplasmosis, for which pathogen-specific management during pregnancy may be considered according to current recommendations [11,12,28].

Another noteworthy finding is the limited implementation of preconception screening for infectious diseases. Fewer than one-fifth of the women had undergone serological evaluation before pregnancy despite national recommendations encouraging assessment of immunity against several vaccine-preventable or vertically transmitted pathogens. Although preconception screening was not associated with earlier referral in the present study, its low uptake highlights an important gap in preventive reproductive healthcare. Strengthening preconception counseling and improving awareness of maternal susceptibility could facilitate vaccination, targeted preventive interventions, and earlier identification of women requiring close surveillance during pregnancy [20,25,26].

Our findings should be interpreted considering current international recommendations [1,2,3,4,5,6,7,8]. Canadian guidelines [28,29,30,32] favor targeted testing based on maternal risk, clinical findings, and epidemiology, avoiding unnecessary investigations that may increase false positives, maternal anxiety, and unwarranted interventions.

In contrast, Italy [22,23,24] and other European countries have long used structured antenatal screening surveillance for selected infections, particularly Toxoplasma gondii and rubella. However, our findings show that specialist referral occurred across all trimesters of pregnancy. The clinical implications of referral timing could not be evaluated in the present cohort.

These differences support further evaluation of prenatal infectious disease pathways. Population-based prevention and individualized risk assessment may be complementary approaches, but their relative effectiveness cannot be determined from the present cohort [9,10,28].

Pediatric infectious disease input may be useful when continuity from maternal infection to fetal assessment and neonatal follow-up is required [12,13,14,36,38,39].

Coordination between prenatal and pediatric services may facilitate continuity of follow-up and recognition of late manifestations of congenital infection, although these outcomes were not specifically assessed in our study [28,29,30,32].

The small sample size and absence of a comparison group preclude assessment of whether the clinical pathway influenced the frequency of congenital infection or other maternal and neonatal outcomes.

**Figure 3:**
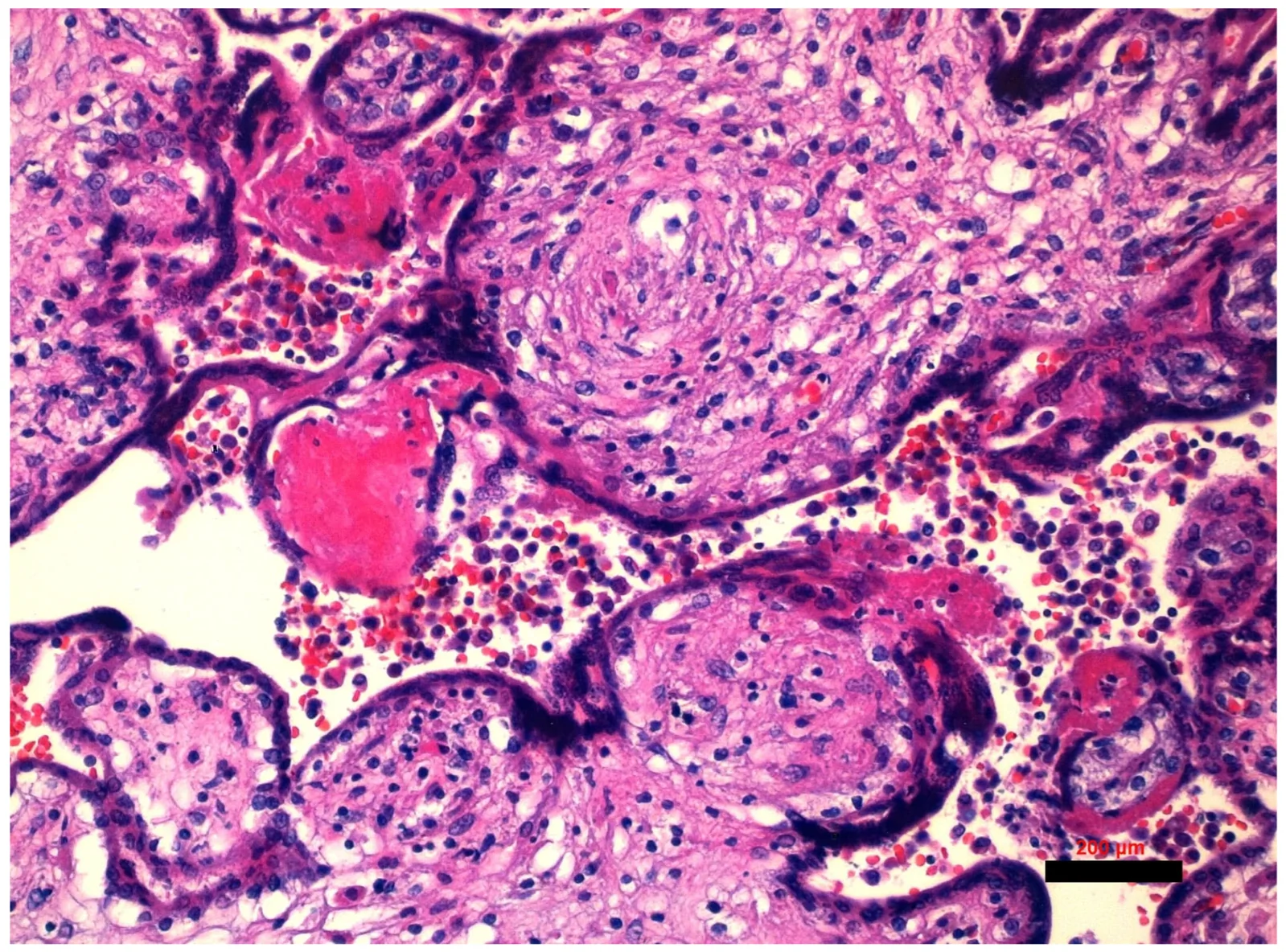
Microphotograph of a placenta with cytomegalovirus (CMV) infection depicting a dense infiltration of the intervillous spaces by plasma cells located at the center of the microphotograph (hematoxylin and eosin staining, 200× magnification).

Only three newborns presented clinical findings compatible with congenital cytomegalovirus infection. Despite the limited number of neonatal cases, placental examination provided additional diagnostic information by complementing maternal serology, microbiological testing, fetal imaging, and neonatal evaluation, thereby strengthening diagnostic confidence in selected pregnancies. Placental histopathology emerged as a valuable complementary component of the diagnostic pathway [40,41]. Although placental lesions are not pathognomonic, histopathological examination combined with immunohistochemistry and PCR may provide valuable confirmatory evidence in selected cases (Figure 3).

### Limitations

The present study provides real-world data for pregnant women referred to a tertiary infectious diseases service and integrates maternal, microbiological, fetal, neonatal, and placental data. Nevertheless, its retrospective single-center design, limited sample size, absence of a comparison group, and incomplete follow-up to delivery for six women limited the causal interpretation and generalizability. The reasons for treatment acceptance or refusal were not systematically collected and therefore remain uncertain. In this retrospective cohort, however, the effect of the referral pathway on diagnostic accuracy, maternal anxiety, or clinical outcomes could not be directly evaluated.

Future prospective multicenter studies should evaluate whether standardized referral pathways affect diagnostic classification, treatment uptake, and maternal or neonatal outcomes.

## 5. Conclusions

Maternal TORCH-related infections remain an important challenge in prenatal care because of their potential fetal and neonatal consequences. In this retrospective tertiary-center cohort, most referrals were prompted by uncertain serological findings, and CMV and *Toxoplasma gondii* accounted for most confirmed maternal infections. The observed difference in treatment uptake between CMV and toxoplasmosis should be interpreted cautiously because the reasons for accepting or declining treatment were not directly assessed. Specialist referral may support interpretation of pathogen-specific investigations, counseling, and individualized clinical decision-making; however, the present design does not establish that this approach improves diagnostic accuracy or maternal and neonatal outcomes. Prospective multicenter studies are required to evaluate these potential benefits and to identify factors associated with timely referral and treatment uptake.

## Figures and Tables

**Table 1:** Pathogen-specific diagnostic investigations, criteria for confirmed maternal infection, and fetal assessment in suspected TORCH infections.

Pathogen	Initial Maternal Investigations	Criteria for Confirmed Maternal Infection	Assessment of Fetal Infection, When Indicated
*Toxoplasma gondii*	Anti-*Toxoplasma* IgG and IgM	IgG seroconversion during pregnancy or a diagnosis of recent primary infection established by a reference laboratory based on IgG/IgM results, IgG avidity, and, when required, follow-up serological testing. Isolated IgM positivity or low IgG avidity alone was considered insufficient.	PCR on amniotic fluid col lected after 18 weeks of gestation and at least 4 weeks after suspected maternal infection.
*Treponema pallidum*	Treponemal test (TPHA) and quantitative RPR or VDRL	Reactive treponemal and non-treponemal tests, interpreted together with the quantitative titer, clinical history, and any previous treatment, to distinguish current infection from previously adequately treated infection.	Fetal and neonatal evaluation according to clinical findings and maternal treatment history.
Rubella virus	Rubella IgG and IgM	IgG seroconversion during pregnancy or a compatible serological profile supported by IgM and low IgG avidity, interpreted in relation to exposure, symptoms, and vaccination history. Isolated IgM positivity was insufficient.	RT-PCR when clinically indicated and feasible.
Cytomegalovirus (CMV)	CMV IgG and IgM	IgG seroconversion during pregnancy or positive CMV IgM together with positive IgG and low IgG avidity, interpreted in relation to gestational age and sampling time. Isolated IgM positivity was insufficient to confirm primary maternal infection.	PCR on amniotic fluid after 21 weeks of gestation and at least 6 weeks after presumed maternal infection.
Herpes simplex virus (HSV-1/HSV-2)	Clinical assessment and examination of lesions	Compatible lesions with HSV DNA detected by PCR from a lesion specimen; in suspected disseminated disease, compatible illness with HSV DNA detected from an appropriate clinical specimen. Serology alone did not establish a new maternal infection.	Assessment according to clinical indications.
Varicella-zoster virus (VZV)	Clinical assessment, VZV IgG, and examination of lesions	Clinically compatible primary varicella supported by VZV DNA detection from a lesion specimen, when available, or documented seroconversion. Pre-existing IgG positivity alone indicated immunity rather than a new infection.	Assessment according to clinical indications.
Parvovirus B19	Parvovirus B19 IgG and IgM	IgG seroconversion during pregnancy or laboratory evidence of recent infection based on IgM and/or maternal blood PCR, interpreted in relation to exposure and sampling time.	Serial fetal ultrasound assessment; additional investigations when indicated.
Hepatitis B virus (HBV)	HBsAg	Confirmed HBsAg positivity, with HBV DNA and other serological markers used to characterize infection. Recent acquisition during pregnancy requires separate evidence, such as documented seroconversion; HBsAg positivity alone does not establish a primary infection.	Neonatal assessment and prevention planning.
Hepatitis C virus (HCV)	Anti-HCV antibodies	Detectable HCV RNA indicating current maternal infection. Recent acquisition during pregnancy requires separate evidence, such as documented seroconversion or a previous negative HCV RNA result. Anti-HCV positivity alone does not establish current infection.	Neonatal follow-up according to applicable recommendations.

Abbreviations: CMV, cytomegalovirus; HBV, hepatitis B virus; HCV, hepatitis C virus; HSV, herpes simplex virus; IgG, immunoglobulin G; IgM, immunoglobulin M; PCR, polymerase chain reaction; RPR, rapid plasma reagin; TPHA, *Treponema pallidum* hemagglutination assay; VDRL, Venereal Disease Research Laboratory; VZV, varicella-zoster virus.

**Table 2:** Clinical and demographic characteristics of 71 enrolled pregnant patients.

Variable	Percentage/Mean
Pregnant women	71
Age	
Mean ± SD	29.4 ± 5.6
Median (IQR [Q1, Q3])	30.0 [26.0, 33.5]
GA at first ID consultation (weeks)	
Mean ± SD	18.7 ± 9.2
Median (IQR [Q1, Q3])	16.0 [11.0, 25.0]
Trimester of pregnancy	
First trimester	29 (40.8%)
Second trimester	27 (38.0%)
Third trimester	15 (21.1%)
Primigravida women	25 (35.2%)
Preconception TORCH screening	13 (18.3%)
CMV	8 (61.5%)
*Toxoplasma gondii*	2 (15.4%)
Rubella virus	1 (7.7%)
*Treponema pallidum*	1 (7.7%)
Parvovirus B19	1 (7.7%)
History of miscarriage	9 (12.7%)
First trimester	6 (66.7%)
Second trimester	3 (33.3%)
Total number of TORCH suspected infections	71
CMV	37 (52.1%)
*Toxoplasma gondii*	25 (35.2%)
Rubella virus	4 (5.6%)
Varicella-zoster virus (VZV)	2 (2.8%)
*Treponema pallidum*	1 (1.4%)
Parvovirus B19	2 (2.8%)
Accepted amniocentesis test	6 (8.5%)
CMV	6 (100%)
Confirmed maternal infections	43 (60.6%)
CMV	23 (53.5%)
*Toxoplasma gondii*	17 (39.5%)
Rubella virus	0 (0.0%)
Varicella-zoster virus	0 (0.0%)
*Treponema pallidum*	1 (2.3%)
Parvovirus B19	2 (4.6%)
Treatment in 43 confirmed cases	30/43 (69.8%)
CMV	12 (40.0%)
*Toxoplasma gondii*	17 (56.7%)
*Treponema pallidum*	1 (3.3%)
Complete follow-up until delivery	65 (91.5%)
Newborns with signs of infection	3/65 (4.6%)

Note: ID = infectious disease; CMV = cytomegalovirus; GA = gestational age; SD = standard deviation.

**Table 3:** Association between pregnancy trimester and demographic, clinical, treatment, and neonatal outcome variables.

Variable	Trimester of Pregnancy			*p*-Value (Test)
	First Trimester	Second Trimester	Third Trimester	
Pregnant women	29 (40.8%)	27 (38.0%)	15 (21.1%)	
Age	28.6 ± 5.6	30.4 ± 4.9	29.3 ± 6.8	0.49 (A)
Primigravida women	8 (27.6%)	9 (33.3%)	8 (53.3%)	0.23 (C)
Confirmed maternal infection	16 (55.2%)	19 (70.4%)	8 (53.3%)	0.41 (C)
Type of confirmed maternal infection				
CMV	8 (27.6%)	11 (40.7%)	4 (26.7%)	
*Toxoplasma gondii*	7 (24.1%)	8 (29.6%)	2 (13.3%)	
*Treponema pallidum*	0 (0.0%)	0 (0.0%)	1 (6.7%)	0.36 (F)
Parvovirus B19	1 (3.4%)	0 (0.0%)	1 (6.7%)	
Treatment in confirmed cases	9 (31.0%)	16 (59.3%)	5 (33.3%)	0.07 (C)
Complete follow-up until delivery	25 (86.2%)	26 (96.3%)	14 (93.3%)	0.48 (F)
Newborns with signs of infection	2 (6.9%)	0 (0.0%)	1 (6.7%)	0.42 (F)

Note: A = one-way ANOVA; C = chi-square test; F = Fisher’s exact test.

**Table 4:** Descriptive characteristics of women who received and did not receive treatment.

Variable	No Treatment	Received Treatment
Pregnant women	41 (57.7%)	30 (42.3%)
Age	29.5 ± 5.6	29.3 ± 5.7
Gestational age at first consultation	14.0 (11.0, 26.5)	17.5 (13.0, 23.0)
Primigravida women	14 (34.1%)	11 (36.7%)
Confirmed maternal infection	13 (31.7%)	30 (100%)
Type of confirmed maternal infection		
CMV	11 (26.8%)	12 (40.0%)
*Toxoplasma gondii*	0 (0.0%)	17 (56.7%)
*Treponema pallidum*	0 (0.0%)	1 (3.3%)
Parvovirus B19	2 (4.9%)	0 (0.0)
Trimester of pregnancy		
First trimester	20 (48.8%)	9 (30.0%)
Second trimester	11 (26.8%)	16 (53.3%)
Third trimester	10 (23.4%)	5 (16.7%)
Complete follow-up until delivery	35 (85.4%)	30 (100%)
Newborns with signs of infection	3 (7.3%)	0 (0.0%)

## Data Availability

The datasets generated and/or analyzed during the current study are not publicly available because they contain sensitive patient information but are available from the corresponding author on reasonable request, subject to institutional and ethical regulations.

## Supplementary Materials

The following supporting information can be downloaded at: https://www.mdpi.com/article/10.3390/pathogens15101054/s1. Table S1. Comparison of selected Canadian and European recommendations for maternal TORCH-related infections.

## Abbreviations

The following abbreviations are used in this manuscript:

TORCH	Toxoplasmosis, Others, Rubella, Cytomegalovirus, Herpes, Syphilis
ID	Infectious disease
GA	Gestational age
CMV	Cytomegalovirus
ISS	Istituto Superiore di Sanità
CSF	Cerebrospinal fluid
PCR	Polymerase chain reaction
ARNAS	Azienda Ospedaliera di Rilievo Nazionale e di Alta Specializzazione
