# Supplementary material for: TORCH-Related Maternal Infections in a Tertiary Referral Center: Diagnostic Pathways and Prenatal Management in the Context of Canadian and European Recommendations
Source: Pathogens. 2026 Oct 4;15(10):1054. doi: 10.3390/pathogens15101054 (PMC13708694; doi:10.3390/pathogens15101054)
Supplement: Supplementary file 1 [file pathogens-15-01054-s001.zip › pathogens-4500825-supplementary.pdf]

Table S1. Comparison of selected Canadian and European recommendations for maternal TORCH-related infections

Pathogen or infection	Selected Canadian recommendations	Selected European recommendations
CMV	No universal screening; risk-based testing	Increasing discussion of universal screening; targeted testing widely used
Toxoplasmosis	Mainly risk-based prevention and testing	Monthly/periodic serological monitoring in susceptible women in Italy
Rubella	Focus on immunity assessment and vaccination before pregnancy	Similar approach with strong elimination programs
Syphilis	Universal antenatal screening recommended	Universal antenatal screening recommended
HIV/HBV/HCV	Universal antenatal screening	Universal antenatal screening
VZV	Serological assessment in women with uncertain immunity	Similar approach
Parvovirus B19	Testing after exposure or clinical suspicion	Similar risk-based approach

Abbreviations: CMV, cytomegalovirus; HIV, human immunodeficiency virus; HBV, hepatitis B virus; HCV, hepatitis C virus; VZV, varicella-zoster virus.
